# Joseph William Bhore (1878-1960): The Architect Behind the Bhore Committee Legacy

**DOI:** 10.7759/cureus.67405

**Published:** 2024-08-21

**Authors:** Alisha Handa, Sonali G Choudhari, Abhay Gaidhane

**Affiliations:** 1 Department of Community Medicine, Jawaharlal Nehru Medical College, School of Epidemiology and Public Health, Datta Meghe Institute of Higher Education & Research, Wardha, IND

**Keywords:** historical vignette, indian civil service officer, health committee, three-tier health system, universal health coverage, primary health care

## Abstract

Sir Joseph William Bhore was a civil official from India. He pioneered the health survey and development committee, known as the Bhore Committee, which set the direction for India's public health facilities and investments. The chairmanship of the Health Survey and Development Committee, which the British colonial administration formed in 1943, is arguably the most well-known role Bhore has held. The committee was founded in 1943 and produced a thorough report in 1946. The committee's suggestions shaped India's health policy and planning and the course of the country's health care growth.

## Introduction and background

**Figure 1:**
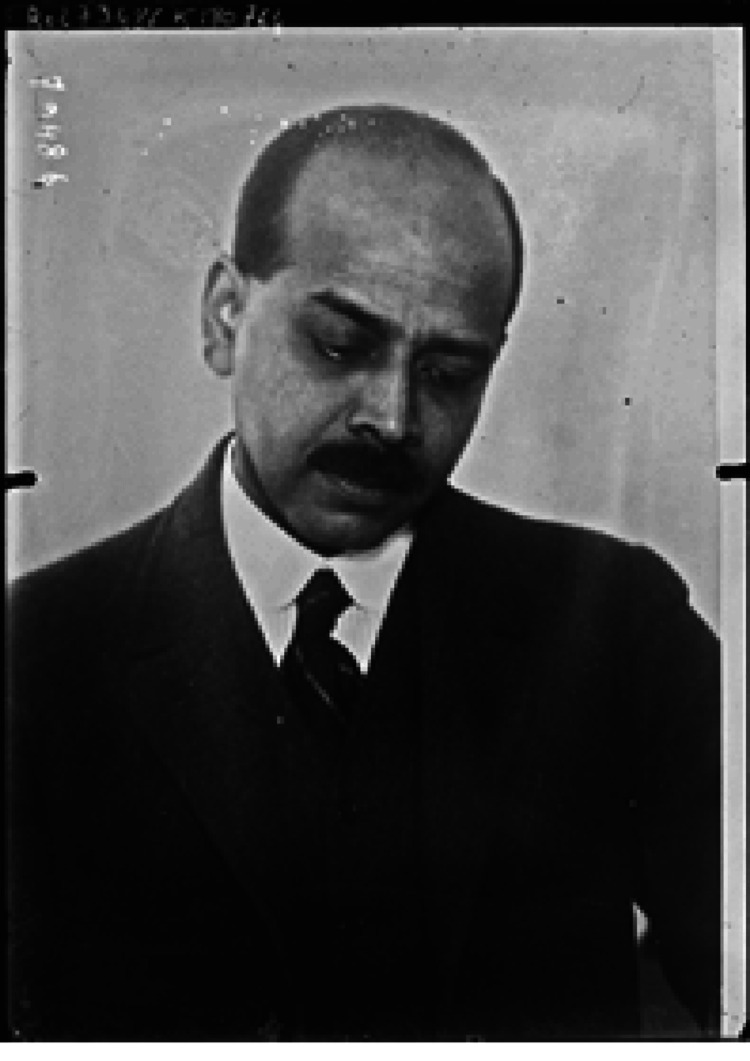
Sir Joseph William Bhore (1922) Source: [2] Copyright/license: The non-commercial reuse of this content is free and open to all.

The key objective of this article is to illustrate Sir Joseph William Bhore's significant contribution to public health in India (Figure 1). In the late 19th century, when Joseph Bhore was born in 1878, India saw substantial socio-political transformation such as the freedom struggle and Indian Independence 1947, Bhore Committee Report 1946, post-World War II reconstruction and planning, and so on. Throughout his career, he held various positions and gained significant experience in public administration and governance, which prepared him for his subsequent contributions to public health. In the “Bhore Committee Report” in 1946, Sir Bhore first introduced us to the idea of comprehensive health care, which would combine curative and preventive medical services within the primary health care system at all administrative levels. In India's public health history, this was much ahead of schedule [1].

## Review

Bhore’s life and career trajectory

Rao Saheb R. G. Bhore's son, Sir Joseph William Bhore, K.C.S.I. (1878-1960), was born in Nasik during the period of British colonization. In 1902, Bhore enlisted in the Indian Civil Service, was given the Madras ICS cadre, and went on to hold several high-level government positions in Cochin and Madras. Throughout his career as a public servant, Bhore held a number of positions in the Departments of Agriculture and Lands (1924-28), Industries and Labor (1930-32), and Commerce and Railways (1932-35). In the UK, he served as India's Acting High Commissioner from 1922 to 1923 and as a member of the Governor General's Executive Council from 1930 to 1932. In 1935, he attended the Silver Jubilee Celebrations in London as a representative of India. Additionally, he served as the Secretary of the Indian Statutory Commission, usually referred to as the Simon Commission, which was founded in 1928 to provide reports on the operations of representative institutions in British India and the Government of India Act 1919 [3]. His early years, schooling, and upbringing were significantly impacted by the political and social setting of the day (specific details of his early life are not widely documented). Bhore's distinguished academic background served as the basis for his outstanding career in public service. He went to Britain to continue his education, as many Indian civil officers of that era did.

He wed Margaret W. Stott in 1911. Margaret W. Stott was awarded several honors, including the Officer of the order of the British Empire. He received his education at University College London, Deccan College in Pune, and Bishop's High School. In 1902, he joined the Indian Civil Service and held several high-level government positions in Madras and Cochin [4].

In his professional journey, one notable aspect was his involvement in health care. As Secretary of the Department of Health Survey and Development Committee India, he was responsible for many public health matters. Being an eminent person on the public health committee, he understood the health care needs and issues in India, specifically in rural India. His works in the public health field are remembered by his efforts in fighting infectious diseases, how he made improvements in the medical services and encouraged sanitation.

Formation of the Bhore Committee

The Government of India announced on October 18, 1943, that the Health Survey and Development Committee, chaired by Sir Joseph Bhore, had been appointed amidst the Second World War following the Quit India Movement [5]. The Bhore Committee was steered by lofty principles, such that rural areas should be the focus of attention and nobody should be denied health services due to an inability to pay [6]. The committee’s task was to examine the nation’s health system and health care providers and make recommendations for future reforms that would strengthen the public health system. The recommendations of the Bhore Committee greatly influenced what happened in the next generation [7].

Objectives and Methodology of the Bhore Committee

The committee conducted a comprehensive survey that included in-depth fieldwork, expert consultations, and statistical analysis of health data. The technique employed was extensive to fully understand the state of health in various communities and areas.

The committee's main recommendations were to integrate preventive and promotional medical health care with establishing primary health centers (PHCs) in rural areas. It further suggested that any project to improve health should prioritize reducing disease and mortality among women and children [8].

The criteria for “comprehensive health care,” as defined by the Bhore committee, were to supply sufficient medical services for promotion, treatment, and prevention, stay as near to those in need as you can, has the broadest collaboration between the public, the profession, and the service, not differentiating on the ability to pay and providing services to all, paying specific attention to the weaker and susceptible population, and creating and keeping homeostasis at home and workplace [9].

The national health plan

Considering India's socioeconomic and health realities, the Bhore Committee set out to accomplish the following goals with their draft plan. There were eight objectives that they aimed to achieve through the plan they formulated. The services should be near the people as much as possible to ensure that the utilization by the community is maximum for which they intend to serve. The Bhore committee's national health plan placed a strong emphasis on the domains of preventative and curative medicine as well as the proactive promotion of good health. The following were the objectives of the plan: to guarantee that the community they are meant to serve makes the most use of them and that there should be a sufficient supply for everyone's medical needs. The location of these preventive and curative care services ought to be as close to the population as feasible. To guarantee that the health program is created soundly, the health organization should provide the broadest platform for public and medical professional engagement. We need to assist the medical and allied professions, including the dental, chemists, and nursing fields. Because of the complexity of modern medical practice, plans must be made for these professionals' representatives to have an impact on the nation's health policy. A variety of consultative, laboratory, and institutional facilities-which together make up "group" practice-must be made available. Special accommodations for certain population segments, such as mothers, children, the mentally ill, and others, will need to be made because they cannot afford it. Everyone should have access to high-quality medical care, both preventatively and curatively, and it is critical to build and preserve a healthy environment in people's homes and other public spaces where they congregate for leisure, enjoyment, or business [7].

Key recommendations of the Bhore Committee

Integrated Health Services

Creating an integrated health services system was one of the Bhore Committee's main recommendations. This approach aimed to deliver seamless medical services that included both preventive and curative measures. The committee emphasized that integration is essential to ensure a continuum of care, reduce redundancies, and increase efficiency.

Primary Health Centers

In India, state-owned PHCs provide health care services in both urban and rural areas. These centers are essentially clinics run by a single physician and are equipped to perform minor procedures. They form the cornerstone of India's publicly funded health system. As of March 31, 2019, India had 30,045 PHCs, with 24,855 located in rural areas and 5,190 in urban areas [8]. The Bhore Committee proposed establishing PHCs as the central hubs for delivering comprehensive curative and preventive health care in rural areas in 1946. The committee advocated for a network of primary and secondary health centers in rural regions to provide residents with easy access to essential health services and medical assistance [10].

Short-term measure: For a population of 40,000, the PHC was suggested as a temporary solution. Each PHC was to employ fifteen additional class IV staff, two sanitary inspectors, two health assistants, one pharmacist, one nurse, four midwives, four public health nurses, four trained dais, and two physicians. The first PHC was established in 1952. Secondary health clinics were designed to offer assistance and supervise and administer PHC activities.

Long-term initiative: The “3 million plan” aimed to regionalize district hospitals with 2,500 beds, secondary health units with 650 beds, and primary health units with 75 beds for every 10,000-20,000 inhabitants [1]. Every detail was covered in the Bhore report: there were 20 beds for infectious disorders, 25 beds for medical issues, 10 beds for surgery, 10 beds for gynecological issues, six beds for malaria, and four beds for tuberculosis. Six medical, surgical, obstetrical, and gynecological experts, 20 nurses, three hospital social workers, eight ward attendants, three compounders, and other non-medical staff were to be assigned to each hospital. With a 650-bed hospital featuring all the major specialties, 140 doctors, 180 nurses, and 178 other staff members (including 15 hospital social workers, 50 ward attendants, and 25 compounders), this primary unit would be connected to the secondary unit, the community health center (CHC). Additionally, each district center was required to establish a 2,500-bed hospital with 269 doctors, 625 nurses, 50 hospital social workers, and 723 other staff members, specializing in tertiary care. Medical colleges would be connected to many of these district hospitals, and all three levels would include resources for medical education and training, such as internships and refresher courses [9].

The Indian Public Health Standards for PHCs include the following goals to provide comprehensive primary health care through PHCs, to attain and maintain a commendable standard of care, and to increase the services' awareness of and adaptability to community needs [11].

Three-Tier Health Care System

**Figure 2:**
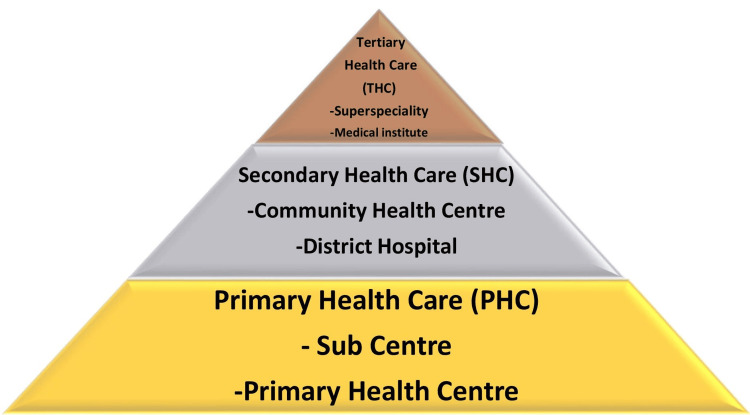
The three-tier health care system given by the Bhore Committee Flowchart credits: Alisha Handa

The Bhore Committee Report (1946) was a ground-breaking document for India and the basis for modern health systems and policies. The current public health care systems were based on suggestions for a three-tiered health care system that would have reduced the need for private practitioners, placed doctors on government payrolls, and provided preventive and curative care in rural and urban areas. This measure was implemented to guarantee that an individual's socioeconomic status would not impede their ability to obtain primary care. Nonetheless, private health care systems emerged concurrently with the incapacity of public health systems to guarantee patients' access to high-quality care, and the quantity of private health care services kept increasing [12]. The system's three tiers are the Subcenter, PHCs, and CHCs (Figure 2).

In 1946, the Bhore Committee reported, "If the loss this nation experiences could be calculated, we believe the results would be so shocking that the entire nation would be awakened and would not sleep until a drastic change had been implemented." This was about the avoidable loss of valuable human capital and the reduction of human efficiency caused by hunger and preventable illness [1].

In summary, the Bhore Committee created the policy framework for a primary, secondary, and tertiary health care system that included precise functioning at every level and scope of activity, along with human resources linked to each level, their functions, controls, and anticipated future results. This created a policy matrix, at last, for a health system that was comprehensive, effective, responsive, and goal-oriented. Throughout the entire attempt, clinical and curative medicine were interwoven with public health. Based on this research, organizational development was then implemented in the states by assigning a single district official, armed with a plethora of equipment, to oversee and keep an eye on every aspect of the health system. Auxiliary nurse midwife (ANM) staffing subcenters at the panchayat level established an organizational framework; in between, the district's block and Tehsil levels housed PHC and CHCs [13].

Medical education reforms

The Bhore Committee's recommendations brought about significant changes in medical education. To address the needs of modern health care, new medical colleges were founded, old ones were renovated, and training programs were updated. As a result of these changes, the medical profession grew and became more competent, necessary to meet the growing needs of public health. Significant modifications were made to medical education as a result of the Bhore Committee's recommendations. New medical colleges were established, existing ones were refurbished, and training curricula were modified to meet the demands of contemporary healthcare. These developments led to the growth and increased competence of the medical profession, which was required to satisfy the expanding demands of public health. The Bhore Committee made significant changes to medical education, including the introduction of a three-month course in social medicine that was used to train doctors and social therapists. Other changes included the creation of PHCs, the expansion of medical schools, and the integration of preventive and curative medicine [7].

Bhore Committee and its importance today

The goal of Ayushman Arogya Mandir is to provide a whole spectrum of health care services, including palliative, curative, rehabilitative, and preventive care, instead of focusing on a limited number of conditions. It consists of two parts that work well together. In order to provide universally accessible, free comprehensive primary health care with an emphasis on wellness and the delivery of a wider variety of services closer to the community, 1,50,000 Ayushman Arogya Mandirs will be established under its first component. The second part is the Pradhan Mantri Jan Arogya Yojana (PM-JAY), which covers more than 10 crore poor and vulnerable households for secondary and tertiary treatment with an annual health insurance benefit of Rs. 5 lakhs. Beyond providing care for mothers and children, Ayushman Arogya Mandirs are intended to offer a wider range of services, such as treatment for non-communicable diseases (NCDs), palliative and rehabilitative care, oral, eye, and ENT care, mental health, and first-rate care for emergencies and trauma. These services will also include free necessary medications and diagnostic services.

Reflection in Light of the Bhore Committee Recommendations

Ayushman Bharat’s structure aligns with the Bhore Committee model of the three-tier system, with health and wellness centers (HWCs) at the primary level, PM-JAY empaneled hospitals (secondary and tertiary levels), and district hospitals integrated into the system. The Ayushman Arogya mandirs provide a blend of preventive, promotive, and curative services as echoed by the Bhore Committee that gave importance to a comprehensive approach towards health care. The HWC essentially target the undeserved and vulnerable rural areas, reflecting the Bhore Committee’s advocacy to prioritize rural health infrastructure [14]

The recommendations of the Bhore Committee focused firmly on the need for a comprehensive public health system. This has laid the foundation for India's primary health care system, which is the backbone of the country's health care delivery system, especially in rural areas [15].

The Bhore Committee focuses on primary health care, and the establishment of PHCs and subcenters in its era remains appropriate and relevant even today. Primary health care must be improved to ensure affordable, equitable, and accessible health care for all citizens [8].

The Bhore Committee emphasized the importance of curative and preventive care. The committee's approach is relevantly essential today, especially for the increasing incidence of NCDs as the curative and preventive care as established by the Bhore committee can help in the burden reduction of NCDs in India [16].

## Conclusions

The field of health care in India has been left with a lasting legacy by Sir Joseph William Bhore and his team. He laid the cornerstone for India's public health system with his advocacies. We still need to create a better modern health care system with good preventive services, community involvement, and primary health care according to Bhore's vision. Even today, the recommendations provided by the Bhore Committee are used as a reference to improve public health care. India is a developing country and is still struggling for universal health and good public health. It is noticeable how salient the concepts developed by Joseph Bhore and his team are for the public health sector of the country.

## References

[1] (2024). Report of the Health Survey and Development Committee. https://nihfw.ac.in/Doc/Reports/bhore%20Committee%20Report%20VOL-1%20.pdf.

[2] (2024). Sir Joseph William Bhore KCSI KCIE CBE (1878 - 15 August 1960). https://gallica.bnf.fr/ark:/12148/btv1b530960667.r=portrait%20Inde?rk=1523612;4.

[3] (2024). Joseph William Bhore. https://en.wikipedia.org/wiki/Joseph_William_Bhore.

[4] Wujastyk D, Smith FM (2010). Modern and global ayurveda: pluralism and paradigms. Asian J Med.

[5] (2024). Report of Health Survey and Development Committee Vol-II. https://nihfw.ac.in/Doc/Reports/Bhore%20Committee%20Report%20-%20Vol%20II.pdf.

[6] Goel S (2007). From Bhore Committee to National Rural Health Mission: a critical review. Int J Health.

[7] Duggal R (1991). Bhore Committee (1946) and its relevance today. Indian J Pediatr.

[8] Behera BK, Prasad R, Shyambhavee Shyambhavee (2021). Primary health-care goal and principles. Healthcare Strategies and Planning for Social Inclusion and Development.

[9] Park K (2015). Park’s Textbook of Preventive and Social Medicine. 23rd Edition. Banarsidar Bhanot K Park: Park's Textbook of Preventive and Social Medicine. Banarsidar Bhanot, Pune.

[10] (2024). The hardest lesson. https://openthemagazine.com/cover-stories/the-hardest-lesson/.

[11] (2024). Indian Public Health Standards (IPHS) Guidelines for Primary Health Centres Revised 2012. https://nhm.gov.in/images/pdf/guidelines/iphs/iphs-revised-guidlines-2012/primay-health-centres.pdf.

[12] Chokshi M, Patil B, Khanna R, Neogi SB, Sharma J, Paul VK, Zodpey S (2016). Health systems in India. J Perinatol.

[13] Patel V, Kumar AK, Paul VK, Rao KD, Reddy KS (2011). Universal health care in India: the time is right. Lancet.

[14] (2024). Ayushman Arogya Mandir. http://hwc.nhp.gov.in/.

[15] Post G (2024). Urgent need for public health practitioners. https://garhwalpost.in/urgent-need-for-public-health-practitioners/.

[16] Srivastava RK, Bachani D (2011). Burden of NCDs, policies and programme for prevention and control of NCDs in India. Indian J Community Med.

